# Boron Clusters Escort Doxorubicin Squashing Into Exosomes and Overcome Drug Resistance

**DOI:** 10.1002/advs.202412501

**Published:** 2024-12-25

**Authors:** Yi‐Ru Bao, Yi‐Jing Chen, Xue‐Fan Deng, Yi‐Ke Wang, Yu‐Xin Zhang, Li‐Li Xu, Wei‐Hua Huang, Shi‐Bo Cheng, Hai‐Bo Zhang, Min Xie

**Affiliations:** ^1^ College of Chemistry and Molecular Sciences Wuhan University 299 Bayi Road Wuhan 430072 P. R. China; ^2^ College of Chemistry and Molecular Sciences Engineering Research Center of Organosilicon Compounds & Materials Ministry of Education and National Demonstration Center for Experimental Chemistry Wuhan University 299 Bayi Road Wuhan 430072 P. R. China; ^3^ Department of Hepatobiliary and Pancreatic Surgery Zhongnan Hospital Wuhan University 169 East Lake Road Wuhan 430072 P. R. China; ^4^ School of Laboratory Medicine Hubei University of Chinese Medicine 16 Huangjia Lake West Road Wuhan 430065 P. R. China

**Keywords:** boron clusters, breast cancer, doxorubicin, drug resistance, exosome

## Abstract

Exosome‐based drug delivery holds significant promise for cancer chemotherapy. However, current methods for loading drugs into exosomes are inefficient and cost‐prohibitive for practical application. In this study, boron clusters are mixed with doxorubicin (DOX) and exosomes, enabling the efficient encapsulation of DOX into exosomes through a superchaotropic effect. Exosomes loaded with DOX and boron clusters (EDB) exhibit superior permeability and the ability to deliver higher concentrations of DOX into DOX‐resistant breast cancer cells. Mechanistic analysis reveals that boron clusters form a supramolecular complex with DOX, which facilitates sustained drug release and effectively inhibits P‐glycoprotein‐mediated DOX efflux. As a result, EDB significantly enhance apoptosis in DOX‐resistant breast cancer cells and suppress tumor growth in cases where DOX alone is ineffective, thereby extending the survival of nude mice. In summary, boron clusters effectively facilitate the incorporation of DOX into exosomes and inhibit DOX efflux, offering a novel strategy to overcome DOX resistance.

## Graphical abstract

Exosomes are promising drug delivery carriers, but their limited loading capacity remains a challenge. This study introduces a novel strategy using boron clusters to enhance doxorubicin loading into exosomes via a superchaotropic effect. This approach enables sustained release and suppresses P‐glycoprotein‐mediated efflux, effectively overcoming resistance in doxorubicin‐resistant breast cancer cells.

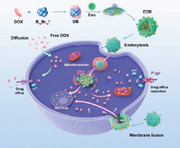

## 1 Introduction

Chemotherapy remains a cornerstone of cancer treatment in clinical practice.^[^ ^1^ ^]^ Doxorubicin (DOX), a well‐established and highly effective anticancer agent, is widely used as a frontline therapy for various types of cancer.^[^ ^2^ ^]^ Despite its proven efficacy, the emergence of drug resistance poses a significant challenge, limiting its long‐term therapeutic effectiveness.^[^ ^3^ ^]^ The primary mechanism of DOX resistance is predominantly linked to the overexpression of P‐glycoprotein (P‐gp), a resistance protein that actively effluxes DOX from cancer cells using adenosine triphosphate (ATP) as an energy source.^[^ ^4^ ^]^ Therefore, developing a novel drug delivery system that inhibits P‐gp‐mediated efflux, enhances the therapeutic efficacy of DOX, and ultimately addresses drug resistance in cancer is of critical importance.

Exosomes, cell‐secreted nanoparticles with a phospholipid bilayer, have gained considerable attention as carriers for transmembrane delivery of DOX due to their low immunogenicity, excellent stability in circulation, and high biocompatibility.^[^ ^5^ ^]^ Moreover, ATP consumption during exosome endocytosis can suppress P‐gp‐mediated DOX efflux through energy depletion.^[^ ^6^ ^]^ These advantages make exosome‐based drug delivery a promising strategy for clinical applications. However, existing methods for loading drugs into exosomes, such as incubation, electroporation, sonication, and freeze‐thaw cycles, are inefficient and face several challenges, including low loading efficiency, compromised exosome integrity, lengthy processing times, and reliance on specialized equipment.^[^ ^7^, ^8^ ^]^ Additionally, developing novel strategies to address DOX resistance beyond exosome‐mediated energy depletion could significantly enhance the efficacy of DOX‐based cancer therapies. Therefore, advancing a more efficient and convenient method for drug loading into exosomes while overcoming DOX resistance has the potential to revolutionize cancer treatment.

Boron clusters, particularly closo‐dodecaborate anions, are renowned for their unique 3D aromaticity and super‐ionization properties. These features enable boron clusters to exhibit superchaotropic effects through a range of weak interactions, including metal coordination, hydrophilic‐hydrophobic interactions, van der Waals forces, and electrostatic interactions with various organic compounds.^[^ ^9^ ^]^ These versatile properties have positioned boron clusters as highly valuable in diverse fields, including biology and chemistry.^[^ ^10^ ^]^ Furthermore, boron clusters have been demonstrated as effective broadband membrane carriers, offering significant potential for pharmaceutical applications.^[^ ^9a,c,d^ ^]^

**Scheme 1:**
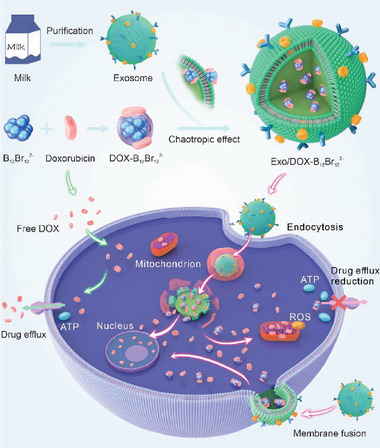
Schematic representation of exosomes loaded with DOX and boron clusters for treating DOX‐resistant breast cancer cells and promoting apoptosis. Boron clusters (B_12_Br_12_^2−^) and DOX form complexes by simple mixing, which then squash into milk‐derived exosomes driven by the superchaotropic effect, resulting in exosomes rapidly loaded with DOX and boron clusters for high capacity. As‐prepared EDB effectively delivers B_12_Br_12_^2−^‐DOX complex into cytoplama of the DOX‐resistant breast cancer cells through membrance fusion and endocytosis of exosomes. The overexpression of P‐glycoprotein (P‐gp) actively pumps DOX out of cells, utilizing ATP to drive this efflux. However, the complex of B_12_Br_12_^2−^‐DOX prevents the binding of P‐gp with DOX, thereby inhibiting P‐gp‐mediated drug efflux and inducing high levels of reactive oxygen species (ROS). As a result, the retained DOX promotes enhanced apoptosis by exerting greater cytotoxic effects on the cancer cells.

In this study, closo‐dodecaborate clusters are employed to enhance DOX loading efficiency into exosomes and address DOX resistance in breast cancer (**Scheme** **1**). The biocompatible dodecaborate clusters, designated as B_12_Br_12_^2−^, are characterized by stable, doubly negatively charged anions and exhibit weak ligating properties. It was revealed that boron clusters form a supramolecular complex with DOX, facilitating the rapid and efficient transport of DOX into exosomes via a superchaotropic effect, which is more superior to commonly employed methods. The resulting exosomes loaded with DOX and B_12_Br_12_^2−^ (designated as EDB) exhibit sustained supramolecular release properties and demonstrate superior capability in delivering higher doses of DOX into DOX‐resistant breast cancer cells, thereby enhancing cell apoptosis. Furthermore, EDB show excellent permeability in tumor spheroids and significantly inhibit tumor growth in cases where DOX alone has lost its efficacy, ultimately prolonging the survival of nude mice. Mechanistic studies reveal that the B_12_Br_12_^2−^‐DOX complex actively inhibits P‐gp‐mediated drug efflux, thereby effectively overcoming DOX resistance in breast cancer.

## 2 Results and Discussion

### 2.1 Fabrication of EDB

Exosomes (Exo) are naturally present in various body fluids, including blood, saliva, urine, and latex, as well as in cellular supernatants, plants, and fruits. In this study, bovine milk was selected as the exosome source due to its wide availability, cost‐effectiveness, and scalability. Additionally, bovine milk consumption is considered safe and nutritionally beneficial. Milk‐derived exosomes exhibit cross‐species tolerance with no reported adverse immune or inflammatory responses, making them an ideal candidate for translational research.^[^ ^11^ ^]^ Exosomes were initially isolated from bovine milk using ultracentrifugation, followed by nanoparticle tracking analysis (NTA) characterization^[^ ^12^ ^]^ (Figure S1, Supporting Information). These exosomes were used as model carriers for preparing therapeutic drug formulations. Three aliquots of Exo were mixed with DOX and B_12_Br_12_^2−^ at molar ratios of 1:2, 1:1, and 2:1, respectively, and incubated for 30 min. Excess B_12_Br_12_^2−^ and DOX were then removed via ultracentrifugation to obtain EDB. The loading efficiencies under these conditions were quantified based on the UV–vis absorption of DOX before and after encapsulation by Exo (Figure S2, Supporting Information). Notably, EDB prepared at a molar ratio of 2:1 (DOX: B_12_Br_12_^2−^) exhibited the highest loading capacity of 27.2%, significantly outperforming the capacities of 14.7% and 17.8% observed at ratios of 1:1 and 1:2, respectively (Figure S3, Supporting Information).

**Figure 1:**
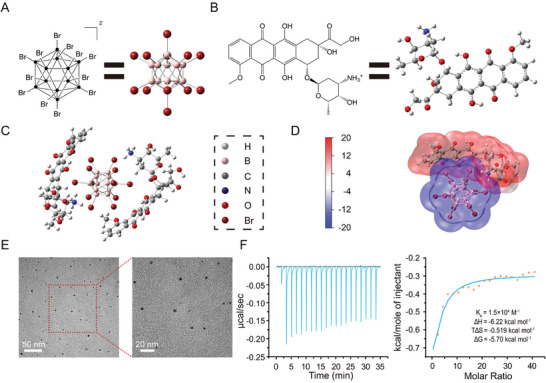
Assembly and structure of DB. The geometries of A) B_12_Br_12_^2−^, and B) DOX. C) Supramolecular structure of the complex, showing one B₁₂Br₁₂^2^^−^ molecule interacting with two DOX molecules. D) Simulated electrostatic potential (ESP) diagram of B₁₂Br₁₂^2^^−^ and DOX (unit: kcal mol^−1^). E) TEM images of supramolecular complex of DOX and B_12_Br_12_^2−^. F) Isothermal titration calorimetry (ITC) analysis, illustrating the direct interaction between B₁₂Br₁₂^2^^−^ and DOX: raw ITC data (left) and apparent reaction heats derived from the calorimetric traces (right).

The differences in loading capacity are attributed to the varying interactions between DOX and B_12_Br_12_^2−^ at different molar ratios. To investigate this, density functional theory (DFT) calculations were performed to optimize the geometries of DOX, B_12_Br_12_^2−^, and their supramolecular complex (DB) using the B3LYP functional and 6–31G(d,p) basis set (**Figure** **1**A–C). An electrostatic surface potential (ESP) map was also generated (Figure 1D). The B_12_Br_12_^2−^ cluster displayed a negatively charged blue surface, while DOX exhibited a positively charged red surface, indicative of electrostatic interactions between the two molecules. At a molar ratio of 1:2 (B_12_Br_12_^2−^: DOX), regular and well‐dispersed nanodots were observed using transmission electron microscopy (TEM) (Figure 1E) and dynamic light scattering (DLS) (Figure S4, Supporting Information). In contrast, at molar ratios of 1:1 and 2:1, electrostatic interactions between the components led to aggregate formation (Figures S4 and S5, Supporting Information), thereby reducing loading efficiency. The binding constant between DOX and B_12_Br_12_^2−^, determined using isothermal titration calorimetry (ITC), was found to be 1.5 × 10⁴  m ^−^¹ (Figure 1F), highlighting the strong interaction that facilitates their rapid aggregation and assembly. Additionally, ITC analysis confirmed the enthalpy‐driven interaction between B_12_Br_12_^2−^ and DOX in solution, indicating non‐canonical membrane translocation mediated by the boron cluster.^[^ ^9a^ ^]^

It has been reported that globular dodecaborate clusters, particularly B_12_Br_12_^2−^, exhibit effects analogous to weakly solvated ions in the Hofmeister series, commonly referred to as the “chaotropic effect.” Notably, the chaotropic properties of B_12_Br_12_^2−^ surpass those of the most chaotropic anions on the Hofmeister scale (ClO_4_^−^, SCN^−^, and PF^6−^), classifying it as superchaotropic in nature. These properties align with those of typical surfactants, given its unique adsorption behavior.^[^ ^13^ ^]^ On a continuous solvation scale, B_12_Br_12_^2−^ begins to mimic the behavior of ionic hydrophobic species, displaying a strong affinity for hydrophobic regions, such as lipid bilayers, and facilitating the direct membrane transport of hydrophilic molecular cargos. B_12_Br_12_^2−^ has been demonstrated to act as an anionic inorganic membrane carrier for a diverse range of hydrophilic cargo molecules, including cationic and neutral peptides, amino acids, neurotransmitters, vitamins, antibiotics, and drugs.^[^ ^9a^ ^]^ The DOX delivery mediated by B_12_Br_12_^2−^ through the superchaotropic effect exhibited enthalpically driven intermolecular interactions, as evidenced by ITC analysis. These findings are consistent with previous reports highlighting the role of boron clusters as versatile membrane carriers.^[^ ^9a^ ^]^

### 2.2 Characterization of EDB

**Figure 2:**
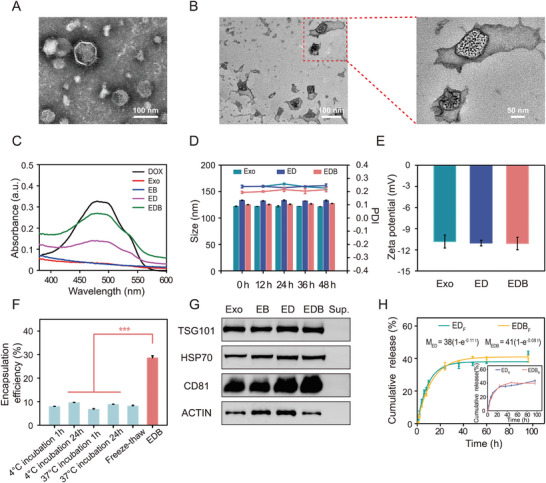
Characterization of EDB. A) TEM image of the Exo; Scale bar: 100 nm. B) TEM images of the EDB; Scale bar: 100 nm; Right: enlarged view of EDB; Scale bar: 50 nm. C) UV–vis spectra of DOX, Exo, EB, ED, and EDB. D) Particle size and PDI of Exo, ED, and EDB in 10% FBS at different times (the lines represent PDI and the bar charts represent size). E) Zeta potential measurements of Exo, ED, and EDB. F) Comparison of DOX loading efficiency into Exo using B₁₂Br₁₂^2^^−^‐based methods versus traditional methods (data are presented as means ± SD from three independent replicates; *p* < 0.05, ^*^ *p* < 0.01, ^**^ *p* < 0.001, and ns indicates no significant difference, analyzed by a two‐sided Student's *t*‐test). G) Western blot analysis of exosomal marker proteins TSG101, HSP70, and CD81 in Exo, EB, ED, EDB, and the supernatant after ultracentrifugation. H) The cumulative release of DOX from ED and EDB in PBS at pH 7.4.

The TEM analysis confirmed the typical vesicle structure of Exo (**Figure** **2**A), and DLS measurements indicated an average diameter of 122.06 ± 0.82 nm for the Exo particles (Figure S6, Supporting Information). Upon the formation of EDB, the integrity of the Exo structure remained intact and was clearly visualized under TEM (Figure 2B). In addition, high‐contrast nanodots, likely representing the complex of B_12_Br_12_^2−^ and DOX, were observed in the EDB but were absent in both pure Exo (Figure 2A) and ED (Exo incubated with DOX alone for 30 min followed by ultracentrifugation; Figure S7, Supporting Information). The UV–vis absorption spectra of EDB revealed a prominent peak at 480 nm, characteristic of DOX, and distinct from pure Exo and EB (Exo mixed with B_12_Br_12_^2−^) (Figure 2C). EDB exhibited a stronger absorption at 480 nm compared to ED, indicating that B_12_Br_12_^2−^ significantly enhanced the efficient loading of DOX into Exo.

The mean diameters of ED and EDB were measured as 133.41 ± 0.76  and 125.10 ± 0.57 nm, respectively, comparable to the size of pure Exo (Figure S8, Supporting Information). When dispersed in 10% fetal bovine serum (FBS) for 48 h, the particle sizes and polydispersity index (PDI) values of Exo, ED, and EDB showed minimal changes, indicating their excellent stability under biological conditions (Figure 2D). The zeta potentials of Exo, ED, and EDB were recorded as −10.82 ± 0.92, −11.03 ± 0.40, and −11.10 ± 0.89 mV, respectively (Figure 2E). The similar negative potentials suggest that DOX was effectively encapsulated within Exo rather than adsorbed onto its surface, and the encapsulation process preserved the biological stability and integrity of Exo.

Compared to conventional methods such as freeze‐thaw cycles and simple incubation (under varying time and temperature conditions, Figure 2F), the incorporation of B_12_Br_12_^2−^ significantly enhanced DOX loading efficiency into Exo. The freeze‐thaw method, a commonly employed approach, achieved a low loading efficiency of 8.2% and resulted in a higher proportion of incomplete Exo (Figure S9, Supporting Information). Similarly, simple incubation of DOX with Exo yielded efficiencies below 10%, with neither extended incubation time nor adjusted temperature improving outcomes. In contrast, the combination of Exo, DOX, and B_12_Br_12_^2−^ achieved a remarkable loading efficiency of 28.6%, while preserving the morphology and structural integrity of Exo (Figure 2B). Western blot analysis confirmed that key exosome markers, including TSG101, HSP70, and CD81, were present both internally and externally in pure Exo, EB, ED, and EDB, demonstrating that neither the incubation process nor the reagents affected the structural or functional integrity of Exo (Figure 2G). The cumulative release profiles of DOX from ED and EDB were analyzed, and the data were fitted to a first‐order dynamic equation (Figure 2H, inset). The release rates for ED and EDB were determined to be 0.11 ± 0.008 and 0.08 ± 0.007 h^−1^, respectively. EDB exhibited a slower release compared to ED, likely due to the restricted diffusion of DOX as a result of its complexation with B_12_Br_12_^2−^ (Figure 2B). This finding highlights the supramolecular sustained‐release characteristics of EDB (Figure 2H).

### 2.3 Evaluation of the Bioactivity of EDB

**Figure 3:**
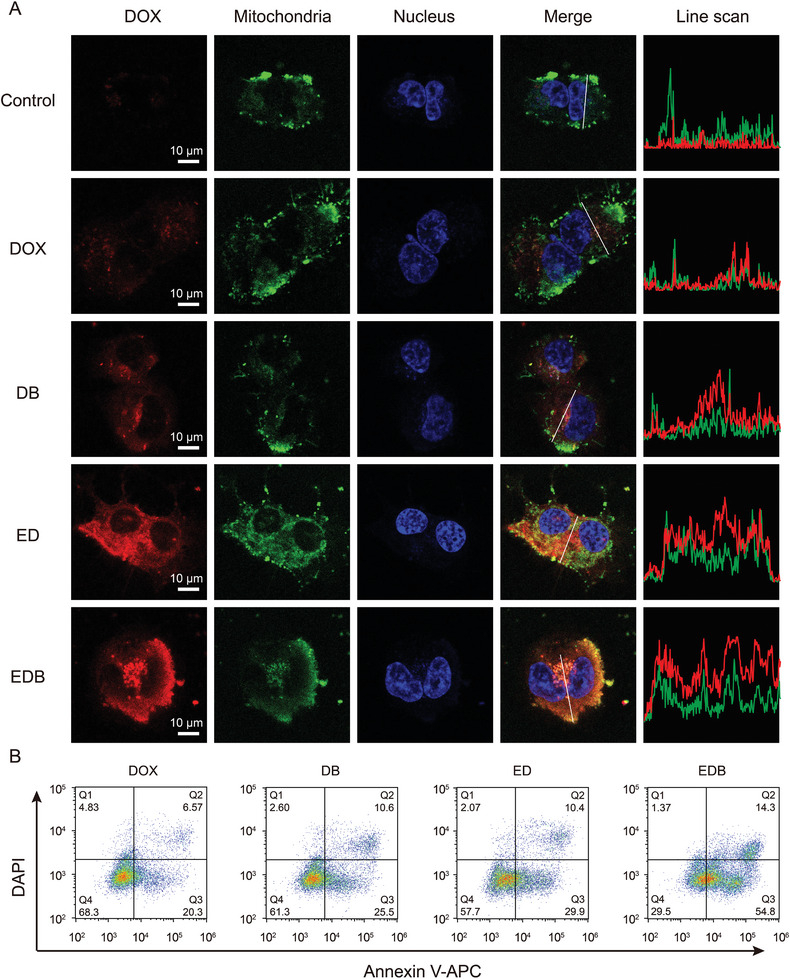
Evaluation of the bioactivity of EDB. A) Confocal images of MCF‐7/DOX cells after 12 h incubation with free DOX, DB, ED, or EDB. Line‐scan fluorescence intensity and quantitative analysis of DOX and mitochondrial signals are shown. Scale bar: 10 µm. B) The flow cytometry of MCF‐7/DOX cells treated with free DOX, DB, ED, and EDB for 48 h, respectively. Q1, necrotic cells; Q2, late apoptotic cells; Q3, early apoptotic cells; Q4, live cells.

To evaluate the bioactivity of EDB, DOX‐resistant MCF‐7 cells (designated MCF‐7/DOX) were incubated with DOX, DB, ED, and EDB, each containing equivalent concentrations of DOX. Confocal microscopy was employed to capture triple‐color fluorescent images, with nuclei stained by Hoechst 33342 (blue), mitochondria labeled by MitoTracker Green (green), and DOX emitting red fluorescence. As shown in **Figure** **3**A, cells incubated with DB and ED exhibited stronger red fluorescence compared to those treated with free DOX. This result suggests that B_12_Br_12_^2−^ serves as an effective carrier for transmembrane drug delivery, attributed to its superchaotropic nature.^[^ ^9a^ ^]^ Exo, widely recognized for their nanoscale size and lipid bilayer structure, demonstrated superior efficiency compared to B_12_Br_12_^2−^ in delivering DOX (Figure 3A).^[^ ^9^ ^]^ Notably, the EDB complex, formed by integrating DOX into Exo with the aid of B_12_Br_12_^2−^, exhibited a markedly enhanced delivery effect compared to the individual components (Figures S10 and S11, Supporting Information). This superiority is attributed to higher DOX loading capacity of EDB compared to ED (Figure 2B). Assuming similar endocytosis capacities in MCF‐7/DOX cells, the internalization of comparable amounts of Exo enabled EDB to deliver significantly more DOX than ED, establishing EDB as the most efficient delivery system. Interestingly, in MCF‐7/DOX cells, DOX was predominantly localized in the cytoplasm and strongly co‐localized with mitochondria, unlike its nuclear localization in normal MCF‐7 cells (Figure S10, Supporting Information). This observation underscores the importance of maintaining a sufficient cytoplasmic concentration of DOX, critical for inducing mitochondrial damage and effectively targeting MCF‐7/DOX cells via a mitochondria‐dependent mechanism. Flow cytometry further confirmed that the percentage of MCF‐7/DOX cells exhibiting DOX fluorescence increased progressively from DOX to DB, ED, and finally EDB (Figure S12, Supporting Information). Collectively, the confocal imaging and flow cytometry results indicate that EDB efficiently enters MCF‐7/DOX cells with high retention.

The bioactivity of DOX, DB, ED, and EDB in inducing apoptosis in MCF‐7/DOX cells was further evaluated using flow cytometry with an Annexin V‐APC/DAPI apoptosis kit. Control experiments confirmed that Exo, B_12_Br_12_^2−^, and their mixture were non‐toxic to MCF‐7/DOX cells (Figure S13, Supporting Information). In contrast, incubation with free DOX caused cellular damage, achieving an apoptosis rate of 26.9%. DB and ED showed improved apoptotic activity, with rates of 36.1% and 40.3%, respectively. Strikingly, EDB exhibited a significantly enhanced apoptotic effect, achieving an apoptosis rate of 69.1% (Figure 3B). These findings were consistent with MTT assay results, which revealed that EDB demonstrated superior dose‐dependent inhibitory effects on MCF‐7/DOX cells compared to DOX and ED (Figure S14, Supporting Information). Moreover, the IC50 value of cells decreased from 30.23 to 23.35 µm with EDB treatment (Table S1, Supporting Information). Scratch wound healing assays further confirmed that EDB effectively suppressed cell migration in MCF‐7/DOX cells, outperforming other DOX‐loaded formulations (Figure S15, Supporting Information). Collectively, these results strongly support the conclusion that EDB significantly inhibits the bioactivity of MCF‐7/DOX cells. Both Exo and B_12_Br_12_^2−^ play pivotal roles in overcoming DOX resistance in MCF‐7 cells, highlighting EDB as a promising therapeutic strategy.

### 2.4 Permeability of EDB in Tumor Spheroids

**Figure 4:**
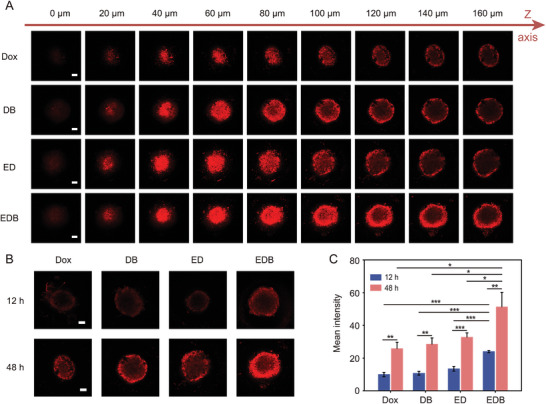
Permeability of EDB in tumor spheroids. A) The penetration of DOX in the tumor spheroids after incubating with free DOX, DB, ED, and EDB for 48 h visualized at interval of 20 µm between the consecutive focal plane; Scale bars: 200 µm. B) CLSM images of MCF‐7/DOX tumor spheroids incubated with free DOX, ED, and EDB for 12 and 48 h, respectively; Scale bars: 200 µm. C) Quantitative mean fluorescence intensity in MCF‐7/DOX tumor spheroids for 12 and 48 h from (B). All data are presented as means ± SD from three independent replicates (*n* = 3). ^*^ *p* < 0.05, ^**^ *p* < 0.01, ^***^ *p* < 0.001, and ns *p* > 0.05 indicate no significant difference, two‐sided Student's *t*‐test.

The permeability of nanodrugs is a critical determinant of their antitumor efficacy. To assess this property, MCF‐7/DOX tumor spheroids were established and separately incubated with free DOX, DB, ED, and EDB. Confocal microscopy images (**Figure** **4**; Figure S16, Supporting Information) revealed that tumor spheroids treated with EDB exhibited the highest levels and most extensive penetration of DOX. Following extended incubation, DOX delivered by EDB diffused from the periphery to the core of the spheroids, emitting intense red fluorescence. This deep penetration was absent in spheroids treated with DB, ED, or free DOX (Figure 4B). Quantitative analysis of mean fluorescence signals further demonstrated that EDB exhibited time‐dependent penetration, significantly surpassing the capabilities of DB, ED, and free DOX (Figure 4C).

### 2.5 Mechanism of EDB in Overcoming DOX‐Resistant Breast Cancer

**Figure 5:**
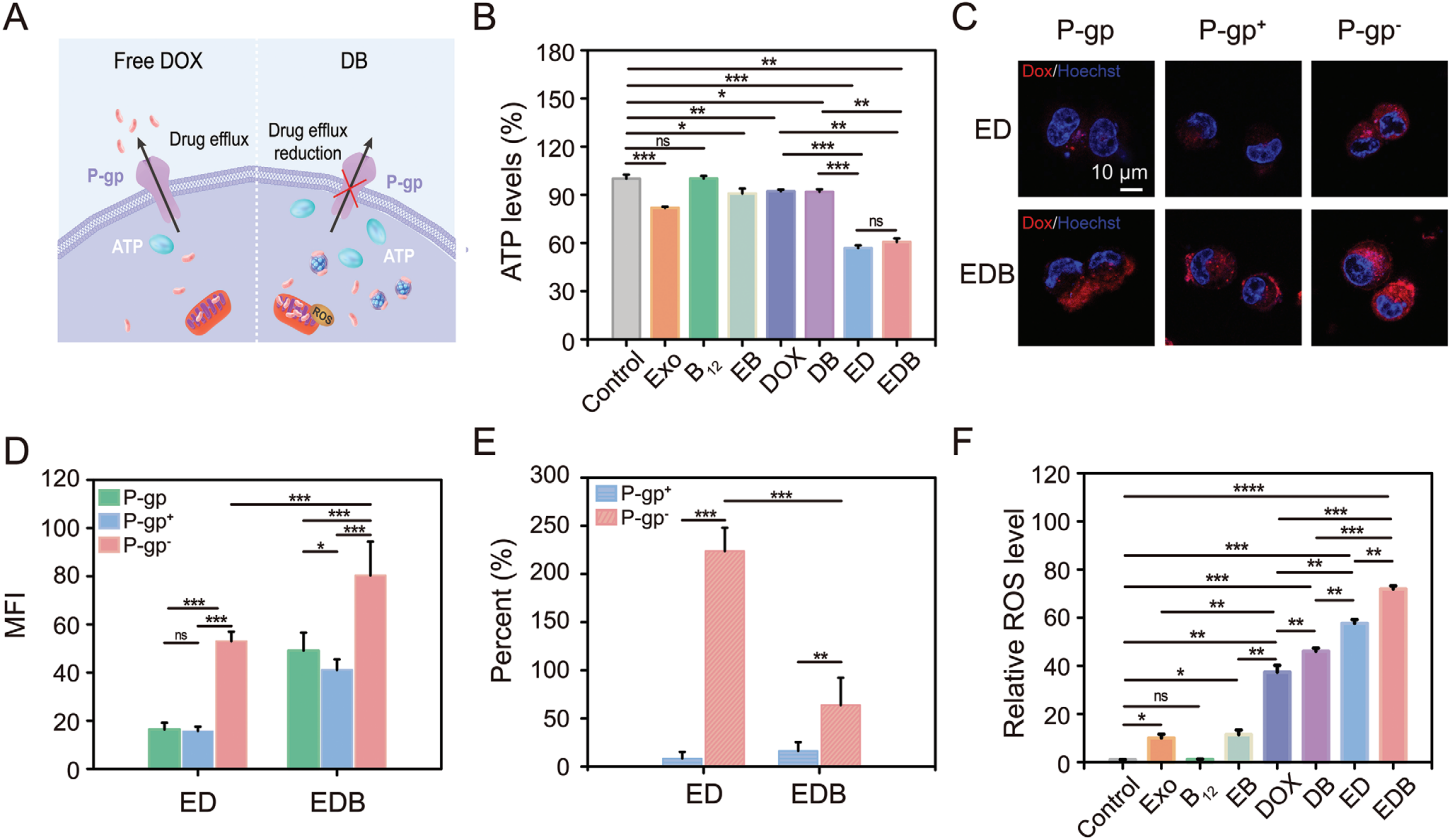
Mechanism of EDB in overcoming DOX‐resistant breast cancer. A) Schematic illustration of the mechanism by that EDB overcomes DOX resistance in breast cancer. B) Relative ATP levels in MCF‐7/DOX cells after being treated with Exo, B_12_Br_12_^2−^, EB, DOX, DB, ED, and EDB for 24 h, respectively. C) Confocal images showing the intracellular uptake of ED and EDB in MCF‐7/DOX cells after stimulation with P‐gp agonist (rifampicin) or inhibitor (verapamil). Scale bars: 10 µm. D) Mean fluorescence intensity of DOX in ED and EDB incubated MCF‐7/DOX cells. E) Variations in mean fluorescence intensity before and after drug (rifampicin and verapamil) stimulation. F) Relative reactive oxygen species (ROS) levels in MCF‐7/DOX cells after being treated with Exo, B_12_Br_12_^2−^, EB, DOX, DB, ED, and EDB for 24 h, respectively. All data are presented as means ± SD from three independent replicates (*n* = 3). ^*^ *p* < 0.05, ^**^ *p* < 0.01, ^***^ *p* < 0.001, and ns *p* > 0.05 indicate no significant difference, two‐sided Student's *t*‐test.

A major mechanism underlying multidrug resistance is the overexpression of the resistant protein P‐glycoprotein (P‐gp), which consumes ATP to expel drugs from cancer cells.^[^ ^3^, ^4^ ^]^ Given this, ATP levels in the cytoplasm are pivotal for maintaining P‐gp function and provide an indicator of drug efflux activity. To investigate this mechanism, ATP levels in MCF‐7/DOX cells were measured after incubation with B_12_Br_12_^2−^, Exo, DOX, EB, DB, ED, and EDB. As shown in **Figure** **5**A, B_12_Br_12_^2−^ alone did not affect ATP levels, whereas both Exo and DOX moderately reduced ATP, likely due to diversified endocytosis and P‐gp pumping activity, respectively. Similarly, ED and DB showed comparable ATP consumption patterns. Based on these observations, it was anticipated that EDB‐treated cells would exhibit lower ATP levels compared to ED‐treated cells due to the synergistic ATP consumption caused by Exo‐induced endocytosis and P‐gp‐mediated DOX efflux.^[^ ^6^ ^]^ Given that EDB loaded a higher amount of DOX into MCF‐7/DOX cells compared to ED, it was expected that the ATP levels in EDB‐treated cells would be lower than those of ED‐treated cells, as this would effectively activate P‐gp to consume ATP for DOX efflux. Surprisingly, ATP levels in EDB‐treated cells were slightly higher than those in ED‐treated cells, although the difference was not statistically significant (Figure 5A).

To explore this unexpected result, P‐gp expression levels were analyzed following treatments with free DOX, DB, ED, and EDB. No significant changes were observed across treatments (Figure S17, Supporting Information), suggesting that the differences in DOX retention were not due to the altered P‐gp expression. Next, P‐gp function was modulated using the agonist rifampicin and the inhibitor verapamil during incubation with ED or EDB. Fluorescence imaging in Figure 5B,C revealed that P‐gp activation did not significantly affect DOX levels in cells treated with either ED or EDB, likely due to the inherently high activity of P‐gp in DOX‐resistant cells. Conversely, P‐gp inhibition substantially increased DOX fluorescence, with a 230% enhancement in ED‐treated cells compared to untreated controls. In contrast, EDB‐treated cells showed only a 60% increase under the same conditions (Figure 5D,E; Figure S18, Supporting Information). These findings suggest that P‐gp was more proficient in expelling DOX delivered via ED compared to EDB, potentially due to the influence of B_12_Br_12_^2−^. The formation of a stable complex between DOX and B_12_Br_12_^2−^ within the exosome (Figure 2B) has been confirmed, rendering the complex resistant to P‐gp‐mediated expulsion upon cellular delivery of EDB. Such complexes are resistant to P‐gp‐mediated expulsion, consistent with previous reports indicating that DOX‐conjugates can evade drug efflux pumps and enhance therapeutic efficacy.^[^ ^15^ ^]^ Additionally, Exo endocytosis reduced intracellular ATP production, further suppressing P‐gp activity and inhibiting DOX efflux.^[^ ^6^ ^]^ Together, these findings suggest that EDB overcomes DOX resistance through a dual mechanism: evading P‐gp‐mediated efflux and reducing ATP levels, thereby enhancing DOX retention and therapeutic efficacy.

Previous studies have shown that DOX induces apoptosis by elevating ROS levels in cancer cells.^[^ ^2^, ^16^ ^]^ To assess this effect, ROS levels in MCF‐7/DOX cells were measured following incubation with B_12_Br_12_^2−^, Exo, DOX, EB, DB, ED, and EDB. As shown in Figure 5F and Figure S19 (Supporting Information), B_12_Br_12_^2−^, Exo, and EB had minimal effects on ROS levels, confirming their cell‐friendly and non‐toxic nature. In contrast, DOX elevated ROS levels, with this effect amplified by both B_12_Br_12_^2−^ and Exo, aligning with the hypothesis that these components enhance DOX delivery and promote ROS generation (Figure 3A). Among the tested formulations, EDB induced the highest ROS levels, suggesting superior intracellular DOX concentrations and enhanced cell‐damaging potential. Thus, EDB effectively overcomes DOX resistance by ensuring efficient drug delivery, inhibiting P‐gp‐mediated drug efflux, and promoting ROS‐mediated apoptosis.

### 2.6 In Vivo Antitumor Efficacy

**Figure 6:**
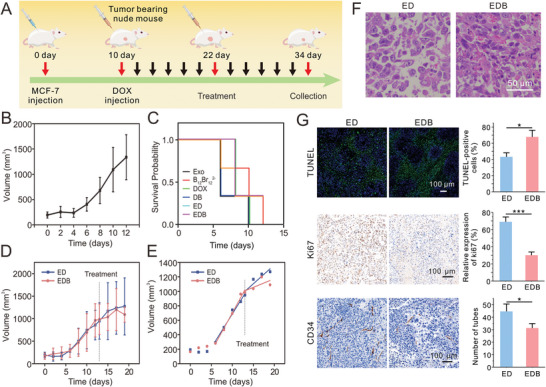
In vivo antitumor efficacy. A) Schematic diagram of the in vivo treatment process in MCF‐7 tumor‐bearing nude mice. B) Tumor volume progression during the DOX‐induced drug resistance process in MCF‐7 tumor‐bearing nude mice. Data are presented as means ± SD (*n* = 18). C) Survival curve of DOX resistant nude mice bearing tumors. D) Tumor volume in nude mice of ED and EDB groups before and after treatment. The results are represented as mean ± SD (*n* = 3). E) Tumor growth curves in nude mice injected with ED or EDB, fitted using a tumor growth model. F) Representative H&E staining of breast cancer tumors from ED‐ and EDB‐treated groups. Scale bar: 50 µm. G) Representative fluorescent TUNEL staining and immumohistochemical staining for Ki67 and CD34 of breast cancer tumors from ED and EDB treatment groups, as well as the corresponding quantitative results of TUNEL, Ki67, and CD34 in ED and EDB groups, respectively. Scale bar: 100 µm. All data are presented as means ± SD from three independent replicates (*n* = 3). ^*^ *p* < 0.05, ^**^ *p* < 0.01, ^***^ *p* < 0.001, and ns *p* > 0.05 indicate no significant difference, two‐sided Student's *t*‐test.

Efforts to establish a DOX‐resistant breast cancer model in nude mice using MCF‐7/DOX cells were unsuccessful. Consequently, an alternative approach was adopted, as outlined in **Figure** **6**A. BALB/c nude mice were subcutaneously injected with MCF‐7 cells, leading to tumor formation (*n* = 18; day 0 to day 10, Figure 6B). The mice then received intravenous injections of free DOX (2.5 mg kg^−1^) every two days. After 12 days, tumor volumes increased significantly, confirming the development of DOX resistance (Figure 6A). At that stage, the mice were intravenously injected with Exo, B_12_Br_12_^2−^, DOX, DB, ED, and EDB at a DOX dosage of 2.5 mg kg^−1^ once every two days (*n* = 3 per group).

Survival durations were monitored across treatment groups. As shown in Figure 6C, mice treated with Exo, B_12_Br_12_^2−^, DB, or ED succumbed by day 6 of treatment. In contrast, mice in the EDB group survived until day 8, indicating that EDB extended survival in DOX‐resistant mice. H&E staining of major organs (heart, liver, spleen, lung, and kidney) revealed normal morphology, confirming the biocompatibility of the materials and that mortality was due to tumor burden rather than systemic toxicity (Figure S20, Supporting Information).

Comparative analysis of ED and EDB treatments revealed differences in therapeutic efficacy. While initial tumor volumes in the ED and EDB groups were similar, tumors in the EDB group exhibited smaller volumes (1090 mm^3^) than those in the ED group (1272 mm^3^) by the end of treatment (Figure 6D). Tumor growth kinetics, characterized by an initial exponential phase followed by linear growth, were modeled to determine growth rates before and after treatment.^[^ ^17^ ^]^ In the ED group, tumor growth rates decreased from 93.3 to 52.0 mm^3^ day^−1^ post‐treatment. Similarly, in the EDB group, growth rates declined from 106.5 to 23.8 mm^3^ day^−1^ post‐treatment, a more substantial reduction compared to ED (Figure 6E). The tumor‐bearing nude mice exhibited a significantly elevated tumor growth rate upon intravenous administration of DOX alone (prior to ED or EDB treatment, the tumor‐bearing nude mice were administered DOX for a duration of 12 days). Upon subsequent ED and EDB treatments, growth rates decreased by factors of 1.8 and 4.5, respectively. EDB exhibited superior tumor inhibition compared to ED. Post‐treatment, tumor excision, and H&E staining confirmed the presence of viable tumor cells (Figure 6F). Additional analyses, including TUNEL assays for apoptosis and immunohistochemical staining of Ki67 (proliferation marker) and CD34 (angiogenesis marker), demonstrated that EDB significantly enhanced apoptosis, suppressed cell proliferation, and inhibited angiogenesis in DOX‐resistant tumors (Figure 6G). These results underscore EDB's therapeutic potential in overcoming drug resistance and inhibiting tumor progression, ultimately improving survival outcomes compared to ED.

## 3 Conclusion

In summary, the boron cluster B_12_Br_12_^2−^ efficiently facilitated the incorporation of DOX into exosomes, forming the EDB complex. Compared to conventional methods of loading DOX into exosomes, this B_12_Br_12_^2−^‐based system offers superior convenience and efficiency. EDB delivered higher concentrations of DOX to DOX‐resistant MCF‐7 cells, effectively suppressing cell migration and inducing apoptosis. It also exhibited excellent penetration into tumor spheroids, demonstrating its capacity to damage solid tumors. Mechanistic analyses revealed that the B_12_Br_12_^2−^ cluster forms a stable supramolecular complex with DOX, enabling sustained release and inhibiting P‐gp‐mediated efflux. Consequently, EDB elevated intracellular ROS levels in MCF‐7/DOX cells, inducing significant cell damage. In vivo, experiments further demonstrated that EDB markedly inhibited tumor growth and prolonged survival in DOX‐resistant nude mice. The combined advantages of exosomes’ transmembrane delivery capabilities and the unique properties of the B_12_Br_12_^2−^‐DOX complex highlight the potential of this system for versatile applications. Given the remarkable benefits of boron clusters and exosome‐based drug delivery, this approach could facilitate the loading of diverse molecules, including cationic drugs, neutral peptides, amino acids, neurotransmitters, vitamins, and antibiotics, into exosomes for transmembrane delivery and the treatment of various diseases.

## 4 Experimental Section

### Animals and Tumor Models

MCF‐7/DOX cell lines were initially used in attempts to construct DOX‐resistant tumor‐bearing nude mice models through various strategies, including subcutaneous injection of MCF‐7/DOX cells alone, MCF‐7/DOX cells mixed with matrix gel, and a mixture of MCF‐7/DOX and MCF‐7 cells in specific proportions. However, these methods were unsuccessful in establishing the desired models. As an alternative, four‐ to six‐week‐old BALB/c nude mice were purchased from Wuhan Myhalic Biotechnology Co., Ltd. (Wuhan, China). MCF‐7 tumor‐bearing nude mice were generated by subcutaneously injecting 107 MCF‐7 cells into the flanks of BALB/c nude mice. All animal experiments were conducted in accordance with ethical regulations for animal research and approved by the Animal Ethics Committee of Wuhan Myhalic Biotechnology Co., Ltd. (Approval No. HLK‐20240308‐001). When the tumor volumes reached 200–250 mm^3^, the mice were intravenously administered free DOX at a dosage of 2.5 mg kg^−1^ every two days. After 12 days, tumor growth persisted, indicating the development of doxorubicin resistance (*n* = 18).

### Statistical Analysis

All data are presented as means ± standard deviation (SD) from three independent replicates (*n* = 3). Statistical significance between experimental groups was evaluated using either a two‐tailed Student's *t*‐test or one‐way ANOVA. Data analysis and visualization were performed using Origin 2018 software and Image J. Significance levels are indicated as follows: *p* < 0.05 (^*^), *p* < 0.01 (^**^), and *p* < 0.001 (^***^); ns denotes no significant difference (*p* > 0.05). All statistical analysis was performed at 95% confidence interval.

## Conflict of Interest

The authors declare no conflict of interest.

## Supporting information



Supporting Information

## Data Availability

The data that support the findings of this study are available from the corresponding author upon reasonable request.
