# Supplementary material for: Boron Clusters Escort Doxorubicin Squashing Into Exosomes and Overcome Drug Resistance
Source: Adv Sci (Weinh). 2024 Dec 25;12(7):2412501. doi: 10.1002/advs.202412501 (PMC11831453; doi:10.1002/advs.202412501)
Supplement: Supplementary file 1 — Supporting Information [file ADVS-12-2412501-s001.docx]

Supplementary Materials

Boron Clusters Escort Doxorubicin Squashing into Exosomes and Overcome Drug Resistance

Yi-Ru Bao,^a†^ Yi-Jing Chen,^a†^ Xue-Fan Deng,^b†^ Yi-Ke Wang,^a^ Yu-Xin Zhang,^a^ Li-Li Xu,^a^ Wei-Hua Huang,^a, c^ Shi-Bo Cheng,^d^* Hai-Bo Zhang,^b^* Min Xie^a^*

[a] Yi-Ru Bao, Yi-Jing Chen, Yi-Ke Wang, Yu-Xin Zhang, Li-Li Xu, Prof. Wei-Hua Huang, Assoc. Prof. Min Xie
College of Chemistry and Molecular Sciences
Wuhan University
299 Bayi Road, Wuhan, P. R. 430072, China
E-mail: [mxie@whu.edu.cn](mailto:mxie@whu.edu.cn)

[b] Xue-Fan Deng, Assoc. Prof. Hai-Bo Zhang
College of Chemistry and Molecular Sciences, Engineering Research Center of Organosilicon Compounds & Materials, Ministry of Education and National Demonstration Center for Experimental Chemistry
Wuhan University
299 Bayi Road, Wuhan, 430072, P. R. China

E-mail: [haibozhang@whu.edu.cn](mailto:haibozhang@whu.edu.cn)

[c] Prof. Wei-Hua Huang
Department of Hepatobiliary and Pancreatic Surgery, Zhongnan Hospital
Wuhan University
169, East Lake Road, Wuhan, 430072, P. R. China

[d] Assoc. Prof. Shi-Bo Cheng
School of Laboratory Medicine
Hubei University of Chinese Medicine
16 Huangjia Lake West Road, Wuhan 430065, P. R. China

E-mail: [shibocheng@hbucm.edu.cn](mailto:shibocheng@hbucm.edu.cn)

† Yi-Ru Bao, Yi-Jing Chen and Xue-Fan Deng contributed equally to this work.

**Table of contents**

1. Supplementary methods4
   1. Materials and instruments4
   2. Quantum computation4
   3. Exosome purification4
   4. Preparation and characterization of DOX-loaded exosome5
   5. Stability of EDB5
   6. Drug release 5
   7. Cell culture 5
   8. Cellular uptake 5
   9. Cell apoptosis assessment 6
   10. Cell viability assay 6
   11. Permeability in tumor spheroids 6
   12. Inhibition of cell migration 6
   13. Western blotting 6
   14. The influence of P-gp activity on cellular uptake7
   15. Intracellular ATP level detection7
   16. Intracellular ROS level detection7
   17. In vivo antitumor efficacy7
   18. Histopathological Evaluation8
2. Supplementary figures9
   1. Figure S1. NTA analysis of exosomes9
   2. Figure S2. Standard curve of DOX based on the absorbance at 480 nm10
   3. Figure S3. Quantification of the encapsulation efficiencies11
   4. Figure S4. DLS measurement of the mixtures of DOX and B_12_Br_12_^2-^12
   5. Figure S5. TEM images of DOX and B_12_Br_12_^2-^ 13
   6. Figure S6. The size distribution of Exosomes14
   7. Figure S7. TEM images of ED15
   8. Figure S8. The size distribution of ED and EDB16
   9. Figure S9. TEM images of Exo loaded with DOX by freeze-thaw 17
   10. Figure S10. Confocal images of DOX, DB, ED and EDB incubated with MCF-7 cells18
   11. Figure S11. Mean fluorescence intensity of DOX in cells19
   12. Figure S12. Flow cytometry of MCF-7/DOX cells20
   13. Figure S13. Flow cytometry results of cell apoptosis21
   14. Figure S14. Viabilities of MCF-7 cells and MCF-7/DOX cells22
   15. Figure S15. Scratch wound healing assays23
   16. Figure S16. Confocal images of DOX permeability in the tumor spheroids24
   17. Figure S17. Western blot analysis of the expression level of P-gp25
   18. Figure S18. Effect of P-gp activity on drug efflux in MCF-7/DOX cells 26
   19. Figure S19. Fluorescent images of MCF-7/DOX stained by DCFH-DA27
   20. Figure S20. H&E staining of excised main organs28
   21. Table S1. IC50 of DOX, ED and EDB for breast cancer cells of MCF-7 and MCF-7/DOX29

**1 Supplementary methods**

**1.1 Materials and instruments**

Fresh milk was purchased from Bright Dairy Co., Ltd (Shanghai, China). The boron clusters (Cs_2_B_12_Br_12_) were generously supplied by Zhang Haibo’s group of Wuhan University College of Chemistry and Molecular Sciences. Doxorubicin (DOX) was purchased from Aladdin Industrial Co., Ltd. (Shanghai, China). RIPA lysis buffer, mouse anti-human TSG101 antibody (TSG101), rabbit anti-human CD81 antibody (CD81), mouse anti-human HSP70 antibody (HSP70), rabbit anti-human Actin antibody (Actin), HRP-labeled goat anti-rabbit secondary, HRP-labeled goat anti-mouse secondary antibody were purchased from Servicebio. Anti-P-gp primary antibody was purchased from Proteintech Group Inc. Anti-rabbit IgG (H+L) (DyLight 800 4X PEG Conjugate) was obtained for Cell Signaling Technology (CST). DOX-resistant MCF-7/DOX breast cancer cells were purchased from iCell Bioscience (Shanghai, China). Human breast cancer cells (MCF-7) were liberally provided by Central South University Xiangya School of Medicine. RPMI 1640 culture medium, DMEM culture medium, fetal bovine serum (FBS), penicillin-streptomycin for cell culture were obtained from GIBCO (USA). Hoechst 33342, MTT cell proliferation and cytotoxicity assay kit, enhanced ATP assay kit, reactive oxygen species assay kit and MitoTracker Green were purchased from Beyotime (Shanghai, China). Annexin V-APC/DAPI apoptosis kit was purchased from Elabscience (Wuhan, China). Matrigel® Matrix Basement Membrane was obtained from Corning (USA).

Unless otherwise noted, all other chemicals with analytical grade were obtained from Sinopharm Chemical Reagent Co., Ltd. (Shanghai, China). Deionized water (Millipore Inc., U.S.A., 18.0 MΩ·cm) was used through the whole experiment.

High-speed centrifuge (Hunan Hengnuo Instrument Equipment Co., Ltd) and ultra-high-speed centrifuge (Beckman Coulter Co., Ltd) were used in the process of extracting exosomes. The size distribution and Zeta potential of the exosomes were measured by dynamic light scattering (DLS) using a Zetasizer Ultra (Malvern Panalytical, UK). Transmission Electron Microscope (TEM) images were taken by a transmission electron microscope (JEM-2100, JPN). The UV–vis absorption spectrum was obtained by a UV–vis spectrophotometer (Agilent, Cary 300, China). The absorbance intensity was obtained through a microplate reader (SYNERGY H1, USA). Cells were cultured inside a humidified incubator (5% CO_2_ and 37 ℃, HERACELL 150, Thermo Scientific). Laser scanning confocal microscopy images were captured by ZEISS LSM 900. The results of flow cytometry were obtained by flow cytometry (Beckman Coulter Co., Ltd, CytoFLEX LX).

**1.2 Quantum computation**

The geometries of DOX, B₁₂Br₁₂²⁻, and their supramolecular complex (DB) were optimized using density functional theory (DFT) with the B3LYP functional and a 6-31G(d,p) basis set. Dispersion corrections were included using the DFT-D3 method. All DFT calculations were conducted using the Gaussian 16 program suite, and the electrostatic surface potential (ESP) was visualized with the Visual Molecular Dynamics (VMD) software.

**1.3 Exosome purification**

Milk-derived exosomes (Exo) were isolated via ultracentrifugation. First, fresh milk was centrifuged at 13,000 g for 30 min to remove fat. The supernatant was acidified to pH 4.6 using 2 M HCl to precipitate caseins, followed by centrifugation at 10,000 g for 1 h. The resulting supernatant was filtered through 0.22 µm polyethersulfone (PES) membranes to remove larger particles and centrifuged at 135,000 g for 80 min to pellet the exosomes. The final pellet was resuspended in PBS and stored at −80 °C for future use.

**1.4 Preparation and characterization of DOX-loaded exosome**

B₁₂Br₁₂²⁻ was synthesized following previously reported methods^[1–3]^. A simple, rapid protocol was developed for loading DOX into Exo. Briefly, 40 µM of B₁₂Br₁₂²⁻ and 80 µM of DOX were mixed with the Exo suspension and gently agitated at room temperature to prepare Exo/DOX-B₁₂Br₁₂²⁻ (EDB). The mixture was ultracentrifuged (135,000 *g*, 80 min) to isolate the loaded product.

For comparison, traditional loading methods, including incubation and freeze-thaw cycles, were applied. In the incubation method, Exo were incubated with DOX at either 4 °C or 37 °C for 1–24 h. In the freeze-thaw method, Exo and DOX underwent three freeze-thaw cycles between −80 °C and 37 °C. The Exo/DOX (ED) product was similarly ultracentrifuged and stored at −80 °C. In all loading protocols, the DOX concentration was maintained at 80 µM.

The morphology of Exo was assessed by TEM following negative staining with phosphotungstic acid. The size distribution and zeta potential of Exo and EDB were determined using DLS. Successful DOX loading was confirmed by UV–vis absorption at 480 nm. Triton X-100 was used to disrupt the Exo phospholipid layer and release encapsulated DOX, and loading efficiency was calculated using a standard calibration curve.

**1.5 Stability of EDB**

The stability of Exo, ED, and EDB was evaluated by incubating the samples in 10% FBS for 48 h. At predetermined time points, the size distribution and polydispersity index (PDI) were measured using DLS. Each condition was tested in triplicate, and results were expressed as mean values.

**1.6 Drug release**

The release of DOX from ED and EDB was studied in PBS (pH 7.4). Samples of ED and EDB were sealed in dialysis membranes (MWCO 3500) and immersed in 50 mL of buffer. At predetermined time intervals, aliquots of the dialysate were withdrawn and replaced with an equal volume of fresh buffer. The concentration of DOX in the dialysate was measured using a microplate reader at 480 nm, based on a standard calibration curve.

**1.7 Cell culture**

The MCF-7/DOX cells were cultured in RPMI 1640 supplemented with 10% FBS, and 1% penicillin-streptomycin. To maintain the resistance phenotype of MCF-7/DOX, the culture media was further supplemented with 500 ng/mL DOX. The MCF-7 cells were cultured in DMEM supplemented with 10% FBS, and 1% penicillin-streptomycin. The cells were cultured in a humidified atmosphere with 5% CO_2_ at 37 ℃.

**1.8 Cellular uptake**

MCF-7/DOX and MCF-7 cells were seeded in 35 mm confocal dishes and incubated overnight. Then free DOX, DB, ED and EDB (with DOX concentration of 30 µM) were added to the dishes and incubated for 12 h at 37 °C, respectively. Untreated cells were used as a blank control. After incubation, cells were washed with PBS and stained with Hoechst 33342 and MitoTracker Green for 1 h before imaging with a confocal microscope.

In parallel, MCF-7/DOX cells were seeded in 6-well plates and treated with free DOX, DB, ED, and EDB (30 µM DOX) for 12 h at 37 °C. After washing with PBS, the cells were detached and analyzed by flow cytometry. Untreated cells were used as a negative control for flow cytometry.

**1.9 Cell apoptosis assessment**

MCF-7/DOX cells were seeded in 6-well plates and incubated for 24 h. Cells were then treated with culture medium, Exo, B₁₂Br₁₂²⁻ (15 µM), Exo/B₁₂Br₁₂²⁻ (EB), free DOX, DB, ED, and EDB (30 µM DOX) for 48 h. The amounts of Exo and EB used were equivalent to those in the EDB group. For apoptosis analysis, cells were processed using an Annexin V-APC/DAPI apoptosis kit following the manufacturer’s protocol and analyzed via flow cytometry.

**1.10 Cell viability assay**

MCF-7/DOX and MCF-7 cells were seeded into 96-well plates and allowed to adhere for 12 h. The cells were then exposed to various concentrations of drug formulations (free DOX, ED, and EDB) for 48 h. Cytotoxicity was assessed using the MTT assay according to the manufacturer’s protocol. Briefly, 10 µL of MTT solution (5 mg/mL) was added to each well, followed by a 4-h incubation. Subsequently, 100 µL of formazan solvent was added to each well to dissolve the formazan crystals during an additional 4-h incubation. Finally, absorbance was measured at 570 nm using a microplate reader to determine cell viability.

**1.11 Permeability in tumor spheroids**

A tumor spheroid assay was performed to evaluate penetration of the EDB. MCF-7/DOX cells were mixed with matrix gel in a 1:1 ratio and seeded into ultra-low attachment 96-well plates (Beyotime, China). The cells were cultured in a humidified incubator at 37 °C, with the medium replaced every 3 days. After 7 days of incubation, the tumor spheroids were then incubated with the culture medium, DOX, DB, ED, and EDB. After incubation for 12 or 48 h, the tumor spheroids were washed with PBS and scanned with a laser scanning confocal microscopy. ImageJ software was used to analyze the mean fluorescence intensity of DOX within the spheroids.

**1.12 Inhibition of cell migration**

A scratch assay was conducted to evaluate the inhibitory effects of Exo-based nanodrugs on the migration of MCF-7/DOX cells. Cells were seeded into 6-well plates and cultured for 24 h to achieve 90% confluence. A straight scratch was then made along the central line of each well using a cell scraper. After washing with PBS to remove debris, the cells were treated with culture medium, free DOX, DB, ED, or EDB, ensuring an equivalent DOX concentration across all groups. Following a 24-h incubation, the scratch wounds were imaged using laser scanning confocal microscopy. The wound area and migration rates were analyzed using image processing software.

**1.13 Western blotting**

MCF-7/DOX cells were seeded at a density of 10^6^ cells/well in 6-well plates and incubated at 37 °C for 12 h. After treating with free DOX, DB, ED and EDB (30 µM DOX) for 12 h, the cells were harvested and lysed with 100 µL RIPA buffer containing protease inhibitor (Beyotime Biotechnology, China). The total protein concentration was determined using BCA protein quantification assay kit. Equal quantities of protein were separated by 8% SDS-PAGE and transferred onto PVDF membranes (Millipore, USA). The PVDF membranes were blocked for 2 h and incubated with anti-P-gp primary antibody overnight at 4 °C, followed by incubation with Anti-rabbit IgG (H+L) (DyLight 800 4X PEG Conjugate) for 1 h. The signals were detected using a CLX-2065 imaging system (LI-COR，USA). Western blottings were quantified using ImageJ software.

**1.14 The influence of P-gp activity on cellular uptake**

MCF-7/DOX cells were divided into three groups: untreated (blank), P-gp inhibitor (verapamil, 5 µM), and P-gp agonist (rifampicin, 10 µM). Each group was treated with culture medium, free DOX, DB, ED, or EDB (30 µM DOX) for 12 h. After treatment, the cells were washed with PBS, stained with Hoechst 33342 for 30 min, and imaged using confocal microscopy to assess the cellular uptake of DOX and the formulations under different conditions.

**1.15 Intracellular ATP level detection**

Intracellular ATP levels were quantified using an enhanced ATP assay kit. MCF-7/DOX cells were seeded into 6-well plates and incubated with culture medium, Exo, B₁₂Br₁₂²⁻ (15 µM), Exo/B₁₂Br₁₂²⁻ (EB), free DOX, DB, ED, or EDB (30 µM DOX) for 24 h. The amounts of Exo and EB were standardized to match those in the EDB group. According to the manufacturer’s instructions, cells were lysed and centrifuged at 12,000 × g for 5 min at 4 °C. The supernatant was collected and mixed with ATP detection working solution at a 1:5 ratio (20 µL:100 µL). Luminescence intensity was measured using a microplate reader.

**1.16 Intracellular ROS level detection**

Intracellular ROS levels were assessed using the DCFH-DA probe. MCF-7/DOX cells were seeded into 24-well plates and incubated with culture medium, Exo, B₁₂Br₁₂²⁻ (15 µM), Exo/B₁₂Br₁₂²⁻ (EB), free DOX, DB, ED, or EDB (30 µM DOX) for 24 h. The amounts of Exo and EB were standardized to match those in the EDB group. After incubation, the cells were washed and cultured in RPMI 1640 medium containing DCFH-DA for 30 min. Fluorescence signals were captured using laser scanning confocal microscopy. ImageJ software was used to analyze the mean fluorescence intensity, reflecting intracellular ROS levels.

**1.17 In vivo antitumor efficacy**

Upon confirmation of DOX resistance in MCF-7 tumor-bearing nude mice, the animals were divided into groups and intravenously injected with Exo, B₁₂Br₁₂²⁻, DOX, DB, ED, or EDB at a DOX dosage of 2.5 mg kg^−1^ every two days (n = 3 per group). Control groups were treated with B₁₂Br₁₂²⁻, DOX, or Exo in doses equivalent to those in the respective DB, ED, and EDB formulations. Exosomes used in these experiments were modified with folic acid to enhance tumor targeting. Throughout the treatment, tumor size and survival time were monitored and recorded to evaluate the therapeutic efficacy of EDB in addressing DOX-resistant breast cancer.

**1.18 Histopathological Evaluation**

Tumor tissues and major organs, including the heart, liver, spleen, lungs, and kidneys, were collected from the mice. The tissues were fixed in formalin, dehydrated, embedded in paraffin, and sectioned into 3 μm thick slices. These sections were subjected to hematoxylin and eosin (H&E) staining and immunohistochemical (IHC) analysis for TUNEL, Ki67, and CD34 expression to evaluate histological and pathological changes. All staining procedures were performed following established clinical laboratory protocols. For H&E staining, sections were stained with hematoxylin to highlight nuclei, followed by eosin to stain cytoplasm and extracellular matrix. The stained sections were then dehydrated, mounted with coverslips, and observed under a light microscope to assess morphological features. For Ki67 and CD34 IHC staining, Sections were incubated with specific primary antibodies targeting Ki67 (a marker of cell proliferation) and CD34 (a marker of angiogenesis), followed by horseradish peroxidase (HRP)-conjugated secondary antibodies. DAB (3,3'-diaminobenzidine) chromogen was applied to visualize antigen-antibody binding. After counterstaining with hematoxylin, sections were dehydrated, mounted, and analyzed microscopically. For TUNEL staining, Sections were processed using a terminal deoxynucleotidyl transferase (TdT)-mediated dUTP nick-end labeling (TUNEL) assay to detect DNA fragmentation associated with apoptosis. Fluorescein-labeled dUTP was incorporated into DNA strand breaks, followed by counterstaining with DAPI (4',6-diamidino-2-phenylindole) to label nuclei. The sections were then imaged using a fluorescence microscope for apoptosis analysis.

**References**

1. E. Justus, A. Vöge, D. Gabel, N‐Alkylation of Ammonioundecahydro‐closo‐dodecaborate(1–) for the Preparation of Anions for Ionic Liquids. *Eur. J. Inorg. Chem.* **2008**, 33, 5245-5250.
2. D. Gabel, D. Moller, S. Harfst, J. Roesler, H. Ketz, Synthesis of S-alkyl and S-acyl derivatives of mercaptoundecahydrododecaborate, a possible boron carrier for neutron capture therapy. *Inorg. Chem.* **1993**, 32, 2276-2278.
3. I. B. Sivaev, A. B. Bruskin, V. V. Nesterov, M. Y. Antipin, V. I. Bregadze, S. Sjöberg, Synthesis of Schiff Bases Derived from the Ammoniaundecahydro-closo-dodecaborate(1−) Anion, [B_12_H_11_NH=CHR]^-^, and Their Reduction into Monosubstituted Amines [B_12_H_11_NH_2_CH_2_R]^-^:  A New Route to Water Soluble Agents for BNCT. *Inorg. Chem.* **1999**, 38, 5887-5893.

**2 Supplementary figures**

**2.1 NTA analysis of exosomes**


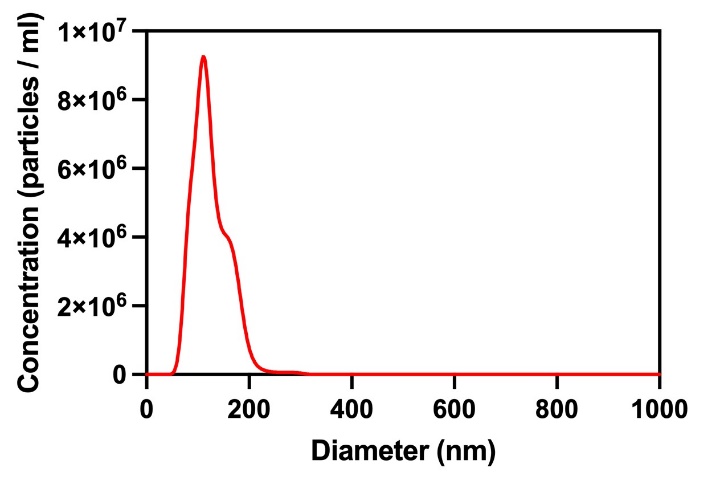


**Figure S1. The NTA analysis of exosomes showed that the mean diameter of milk-derived Exo is 126 nm with the concentration of 6.2×10^12^ exosomes/mL (the exosomes obtained by ultracentrifugation was suspended in 1 mL of PBS and then diluted 10^4^ times for NTA analysis).**

**2.2 Standard curve of DOX based on the absorbance at 480 nm**


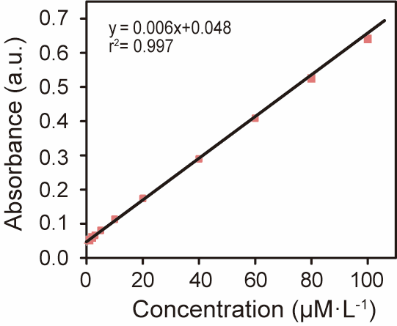


**Fig. S2.** **Standard curve of DOX based on the absorbance at 480 nm.**

**2.3 Quantification of the encapsulation efficiencies**


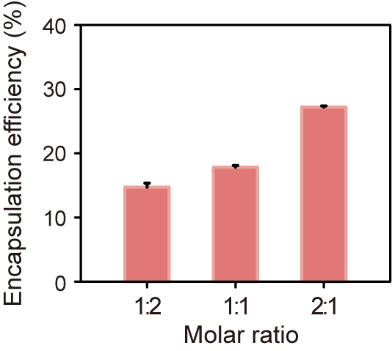


**Fig. S3. Quantification of the encapsulation efficiencies at different molar ratios of DOX to B_12_Br_12_^2-^ (1:2, 1:1, 2:1). Results are represented as mean ± SD (n = 3).**

**2.4 DLS measurement of the mixtures of DOX and B_12_Br_12_^2-^**


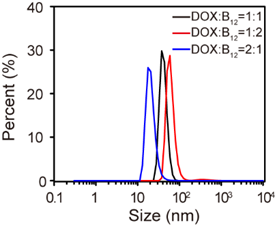


**Fig. S4.** **DLS measurement of the mixtures of DOX and B_12_Br_12_^2-^ under different molar ratios.**

**2.5 TEM images of DOX and B_12_Br_12_^2-^**


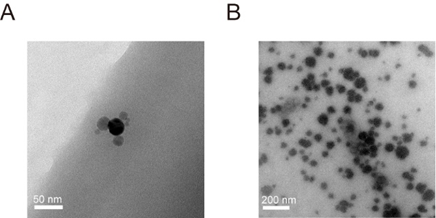


**Fig. S5. TEM images of DOX and B_12_Br_12_^2-^ in molar ratio of 1:1 (A) and 1:2 (B).**

**2.6 The size distribution of Exosomes**


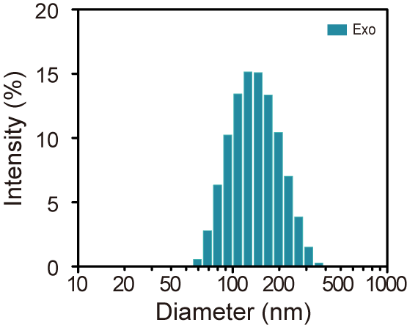


**Fig. S6.** **The size distribution of Exo.**

**2.7 TEM images of ED**


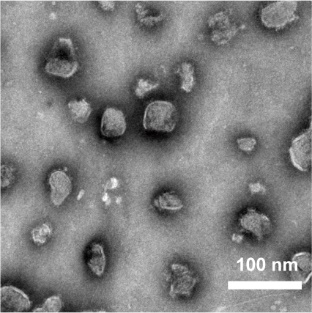


**Fig. S7.** **TEM images of ED.** Exo incubated with DOX for 30 min followed by ultracentrifugation; Scale bar: 100 nm.

**2.8 The size distribution of ED and EDB**


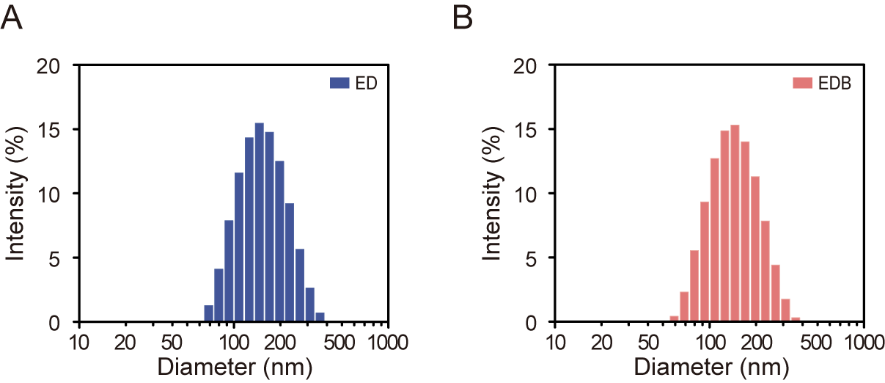


**Fig. S8.** **The size distribution of ED (A) and EDB (B).**

**2.9 TEM images of Exo loaded with DOX by freeze-thaw**


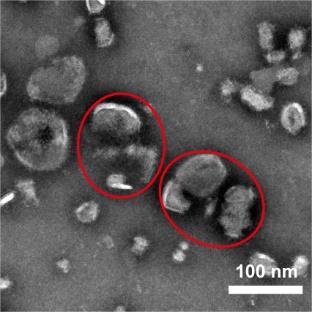


**Fig. S9.** **TEM images of Exo loaded with DOX by freeze-thaw**; Scale bar: 100 nm.

**2.10 Confocal images of DOX, DB, ED and EDB incubated with MCF-7 cells**


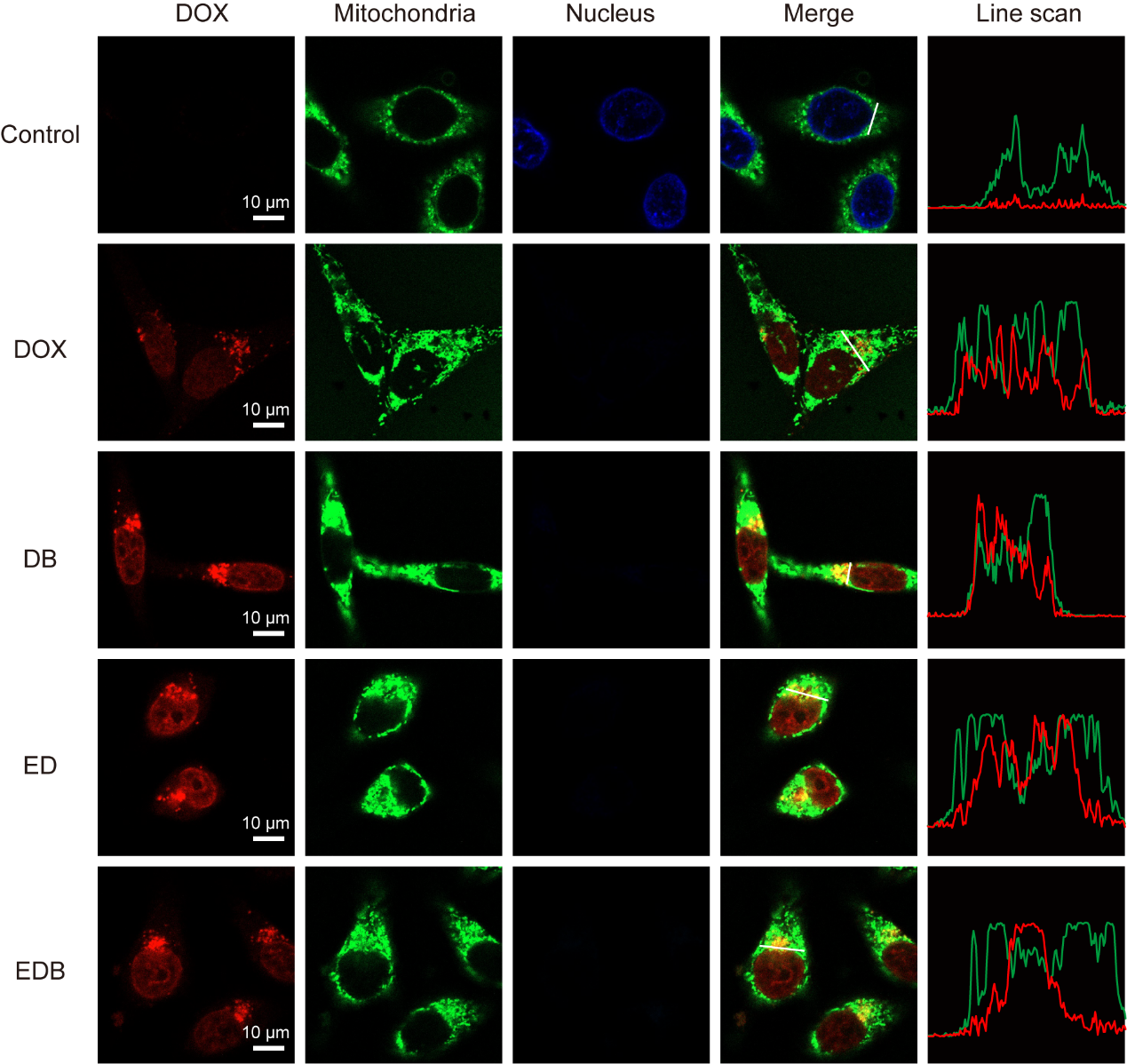


**Fig. S10.** **Confocal images of DOX, DB, ED and EDB incubated with MCF-7 cells for 12 h.** Line scanning is a quantitative assay of DOX and mitochondria in MCF-7 cells. Scale bars: 10 µm.

**2.11 Mean fluorescence intensity of DOX in cells**


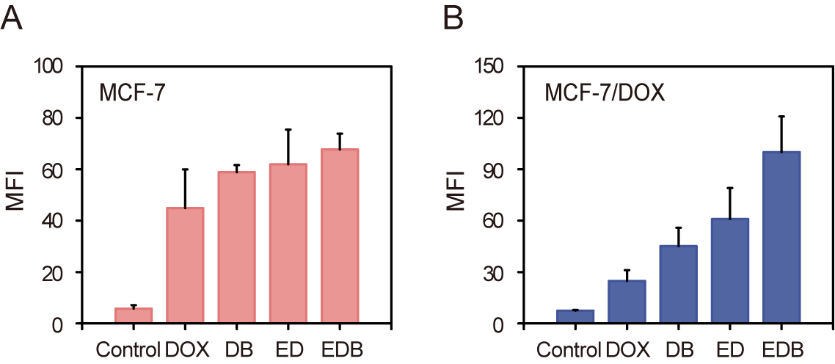


**Fig. S11.** **Mean fluorescence intensity of DOX in cells (A) MCF-7 cells (B) MCF-7/DOX cells. Results are represented as mean ± SD (n = 3).**

**2.12 Flow cytometry of MCF-7/DOX cells**


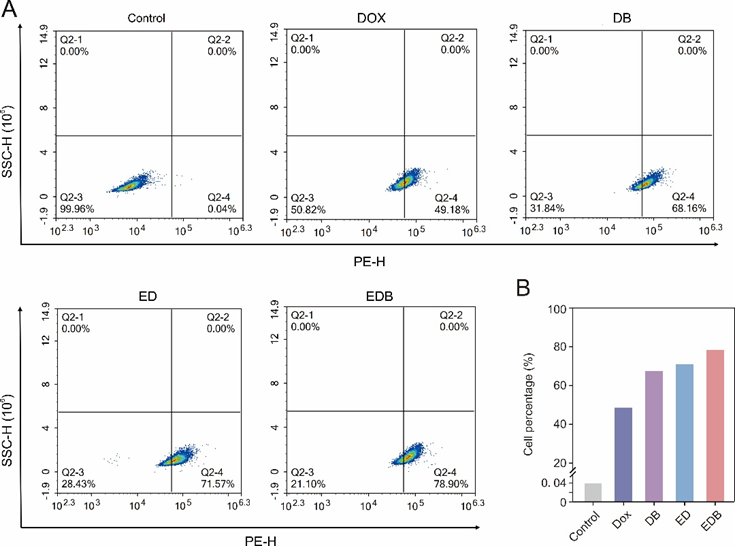


**Fig. S12 Flow cytometry of MCF-7/DOX cells after incubation with free DOX, DB, ED and EDB, respectively, as well as control of MCF-7/DOX without any treatment. A. Scatter diagram, and B. Statistical data.**

**2.13 Flow cytometry results of cell apoptosis**


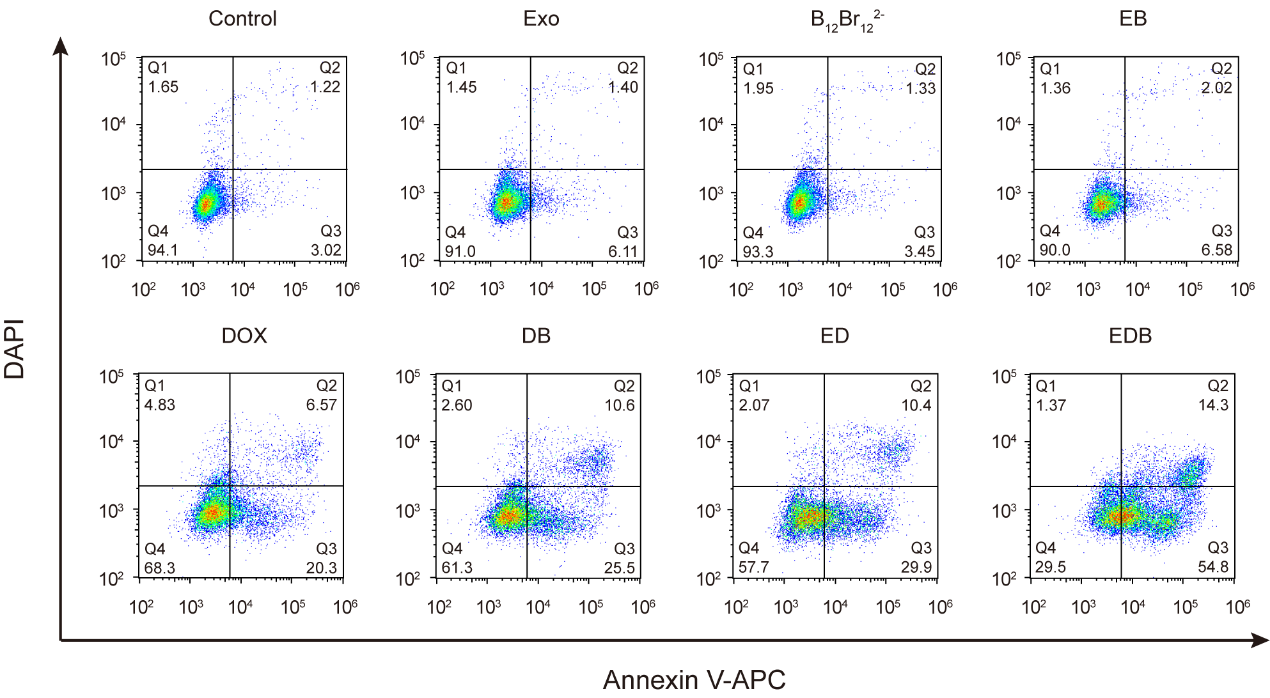


**Fig. S13.** **The flow cytometry results of cell apoptosis of MCF-7/DOX cells treated with free DOX, DB, ED and EDB (30** **µM DOX), and Exo and EB (equivalently to EDB), and B_12_Br_12_^2-^ (15 µM) for 48 h.** Q1, necrotic cells; Q2, late apoptotic cells; Q3, early apoptotic cells; Q4, live cells.

**2.14 Viabilities of MCF-7 cells and MCF-7/DOX cells**


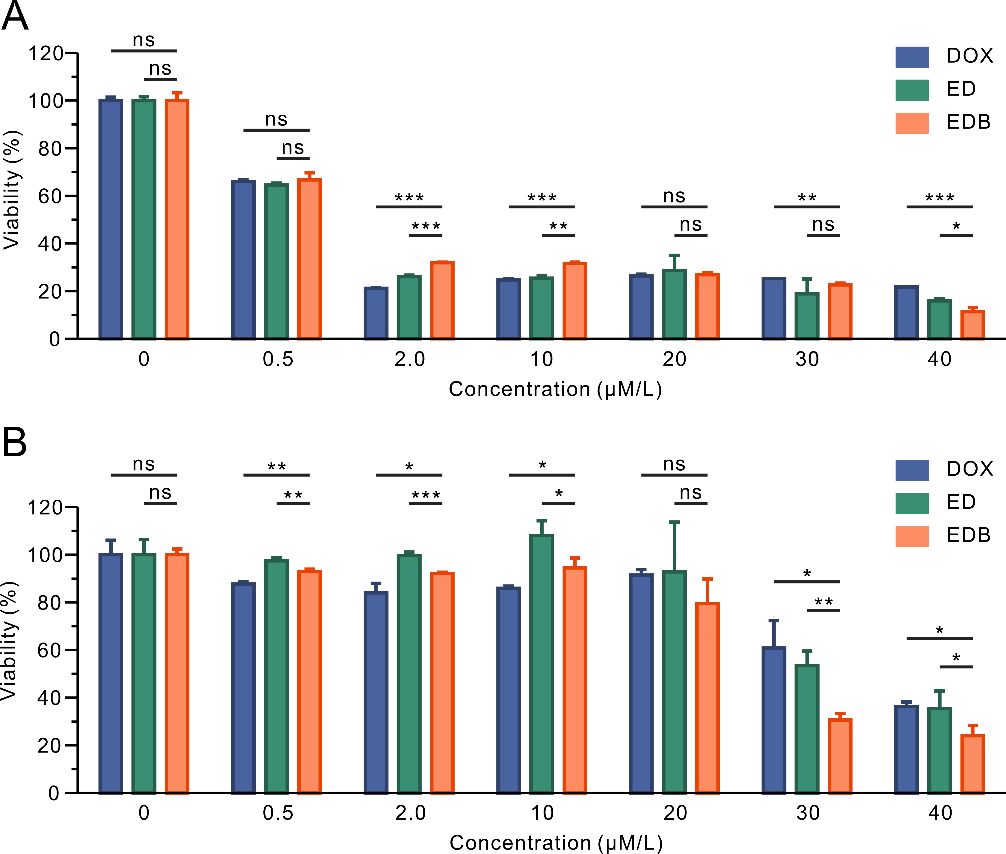


**Fig. S14.** **Viabilities of MCF-7 cells (A) and MCF-7/DOX cells (B) after incubation with different concentrations of DOX, ED and EDB for 48 h.** All data are presented as means ± SD from three independent replicates (n = 3). *p < 0.05, **p < 0.01, ***p < 0.001, and ns p > 0.05 indicate no significant difference. Kruskal-Wallis one-way ANOVA with post hoc Dunn’s test was used to determine the significant difference.

**2.15 Scratch wound healing assays**


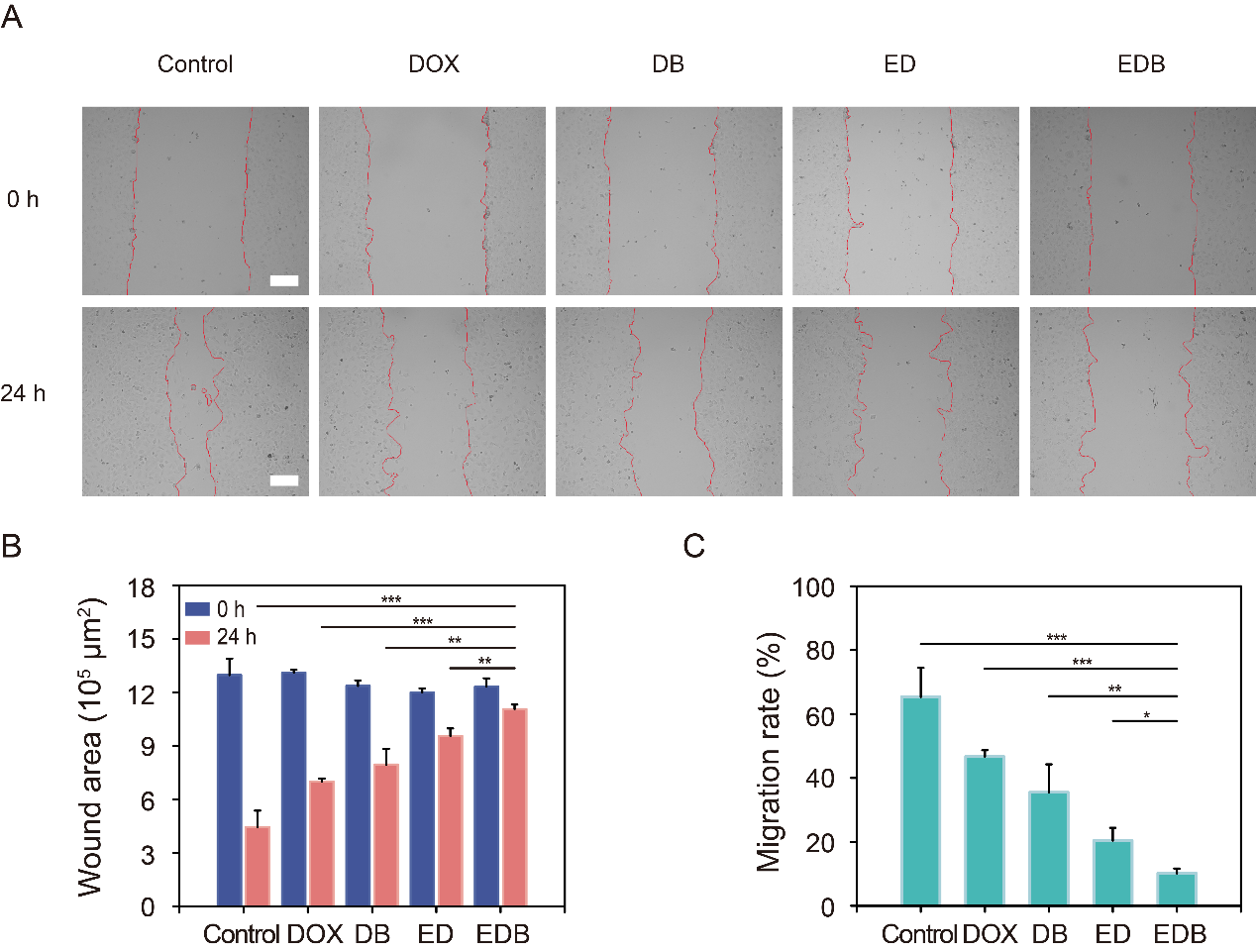


**Fig. S15.** **Scratch wound healing assays.** (**A**) The scratch wound images of MCF-7/DOX cells after incubating with free DOX, DB, ED, EDB for 0 h (top row) and 24 h (bottom row), respectively; Scale bars: 200 µm. (**B, C**) Wound area and migration rate obtained from (**A**). The results represented as mean ± SD (n = 3). *p < 0.05, **p < 0.01, ***p < 0.001, and ns p > 0.05 indicates no significant difference, two-sided Student’s t test.

**2.16 Confocal images of DOX permeability in the tumor spheroids**


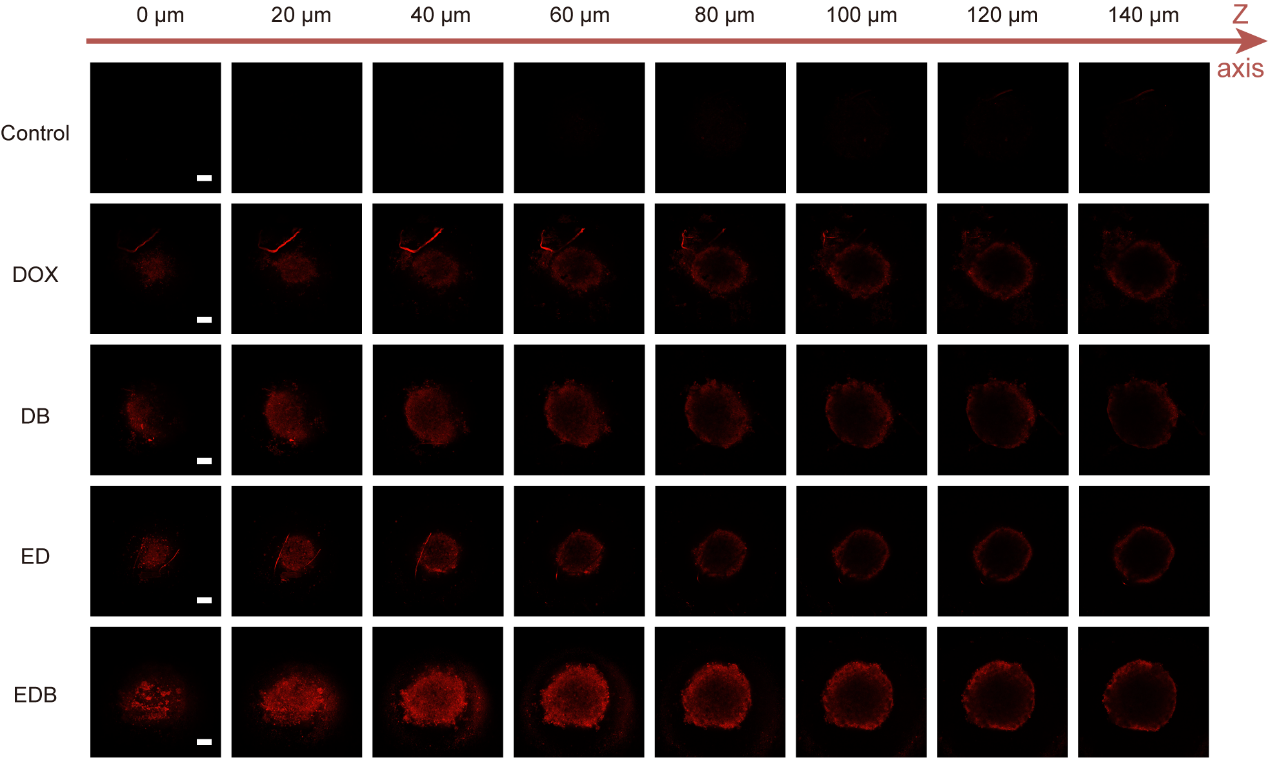


**Fig. S16.** **Multilevel scan of the DOX permeability in the tumor spheroids after incubating with culture medium, DOX, DB, ED and EDB for 12 h by interval of 20 µm between the consecutive focal plane**; Scale bar: 200 μm.

**2.17 Western blot analysis of the expression level of P-gp**


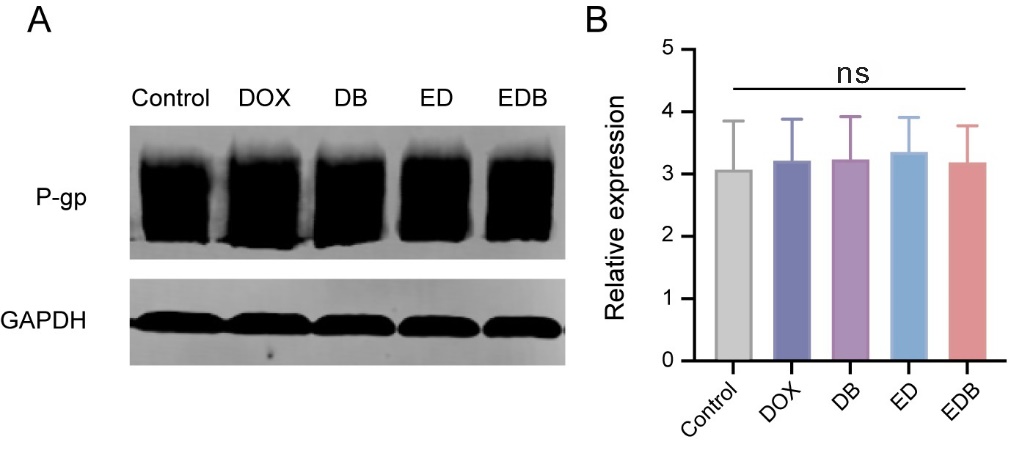


**Fig. S17. Western blot analysis of the expression level of P-gp following treatments with free DOX, DB, ED, and EDB, respectively, as well as control of MCF-7/DOX without any treatment. (A**) Representative images. (**B**) The quantitative results of three parallel samples and tests. The results showed p > 0.05 among each group, indicating no significant difference. Kruskal-Wallis one-way ANOVA with post hoc Dunn’s test was used to determine the significant difference.

**2.18 Effect of P-gp activity on drug efflux in MCF-7/DOX cells**


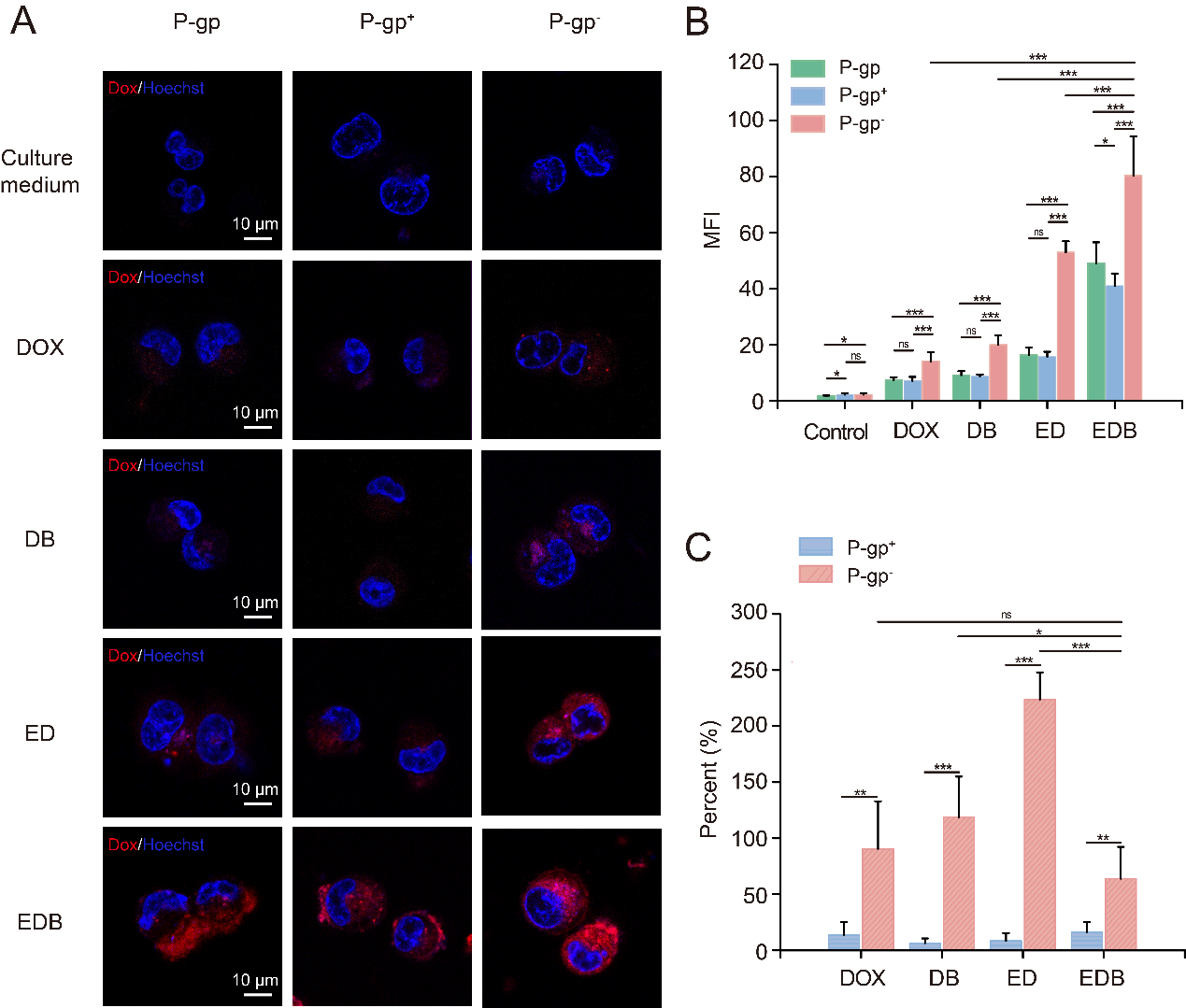


**Fig. S18.** **Effect of P-gp activity on drug efflux in MCF-7/DOX cells.** (A) Effects of P-gp agonist (rifampicin) and inhibitor (verapamil) on DOX, DB, ED, and EDB intracellular uptake; Scale bars: 10 µm. (B) Quantification of the mean fluorescence intensity. (C) Variations in mean fluorescence intensity before and after drug (rifampicin and verapamil) stimulation. The results represented as mean ± SD (n = 3). *p < 0.05, **p < 0.01, ***p < 0.001, and ns p > 0.05 indicates no significant difference, Kruskal-Wallis one-way ANOVA with post hoc Dunn’s test (B) or two-sided Student’s t test (c) were used to determine the significant difference.

**2.19 Fluorescent images of MCF-7/DOX stained by DCFH-DA**


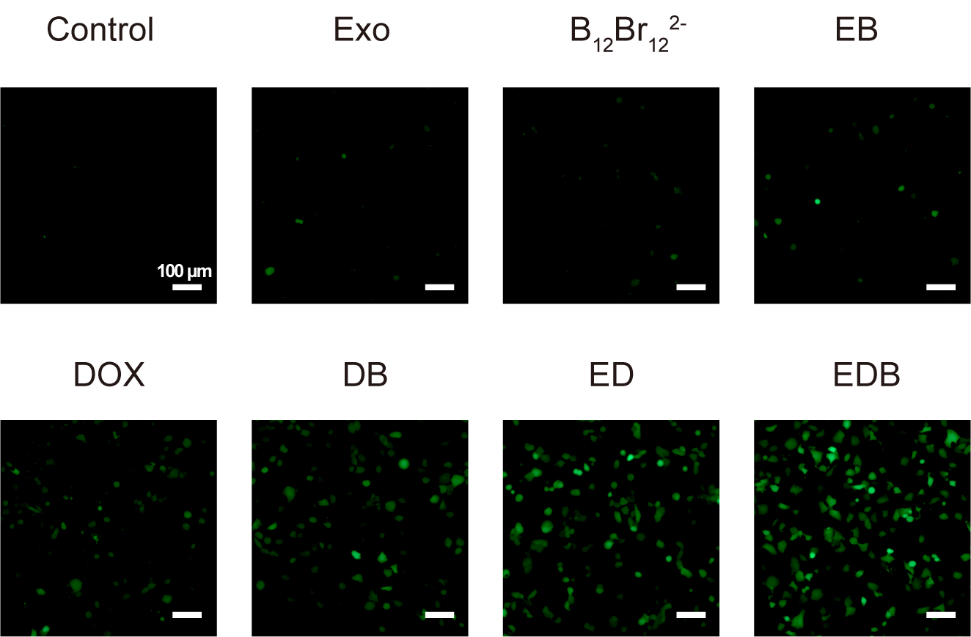


**Fig. S19.** **Fluorescent images of MCF-7/DOX cells after being treated with Exo, B_12_Br_12_^2-^, EB, DOX, DB, ED, and EDB for 24 h and then stained by DCFH-DA**; Scale bar: 100 μm.

**2.20 H&E staining of excised main organs**


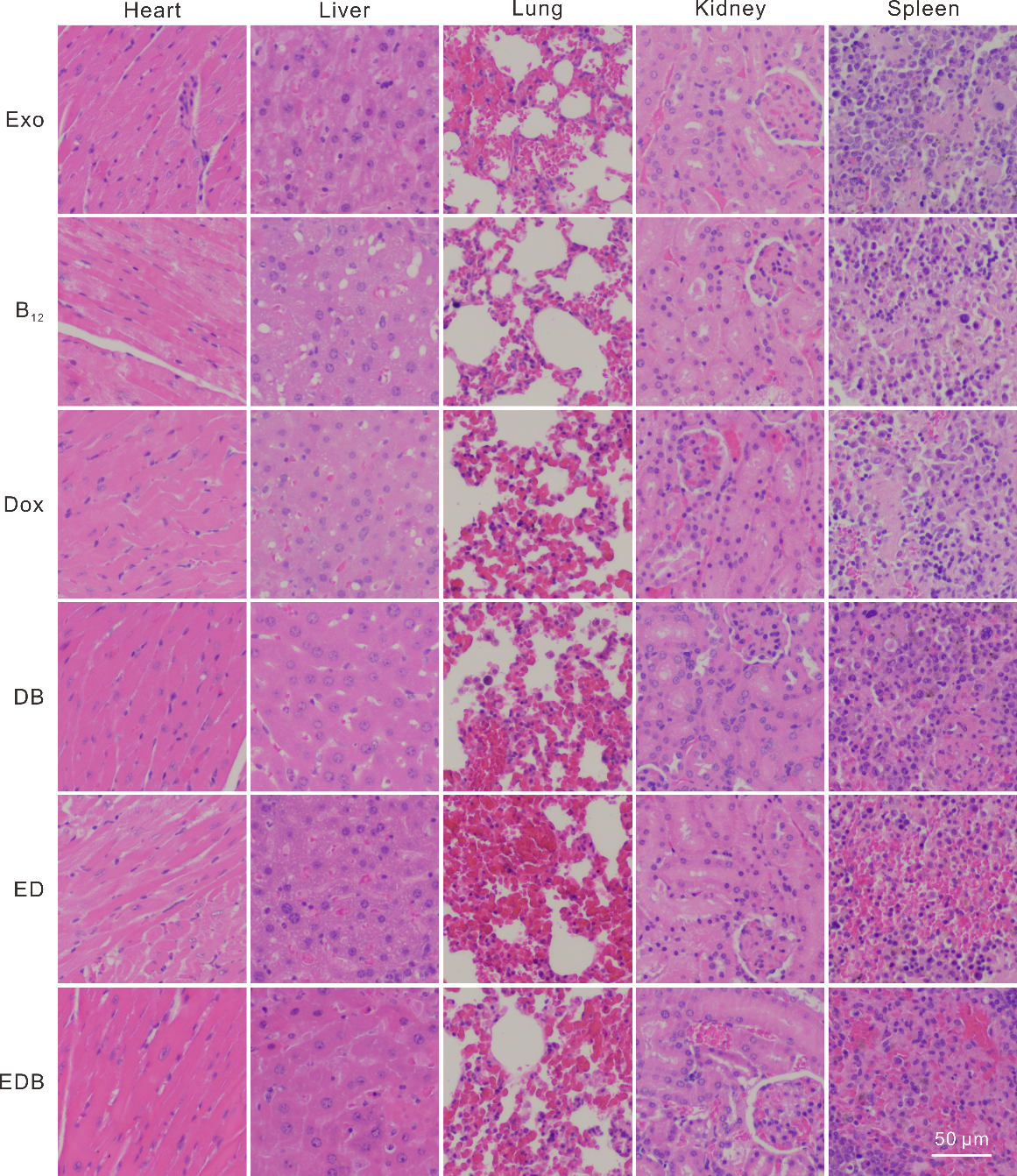


**Fig. S20. H&E staining of excised main organs (heart, liver, lung, kidney, spleen) of mice after treatment by Exo, B_12_Br_12_^2-^, DOX, DB, ED and EDB, respectively.** Scale bar: 50 μm.**Table S1. IC_50_ of DOX, ED and EDB for breast cancer cells of MCF-7 and MCF-7/DOX**.

| Treatments | Cell lines / IC_50_ ± SD (μM) | |
| --- | --- | --- |
|  | MCF-7 | MCF-7/DOX |
| DOX | 0.57 ± 0.06 | 30.23 ± 3.83 |
| ED | 0.37 ± 0.11 | 27.46 ± 3.32 |
| EDB | 0.33 ± 0.21 | 23.35 ± 2.84 |
